# Botulinum toxin-A effects on pain, somatosensory and psychosocial features of patients with refractory masticatory myofascial pain: a randomized double-blind clinical trial

**DOI:** 10.1038/s41598-024-54906-z

**Published:** 2024-02-20

**Authors:** Giancarlo De la Torre Canales, Rodrigo Lorenzi Poluha, Leonardo Rigoldi Bonjardim, Malin Ernberg, Paulo César Rodrigues Conti

**Affiliations:** 1 Division of Oral Rehabilitation, Department of Dental Medicine, Karolinska Institutet, and The Scandinavian Center for Orofacial Neurosciences (SCON), Huddinge, Sweden; 2 Egas Moniz Center for Interdisciplinary Research (CiiEM), Egas Moniz School of Health & Science, Caparica, Almada, Portugal; 3 Department of Dentistry, State University of Maringá, Paraná, Brazil; 4 Bauru Orofacial Pain Group, Department of Biological Sciences, Bauru School of Dentistry, University of São Paulo, São Paulo, Brazil; 5 Bauru Orofacial Pain Group, Department of Prosthodontics, Bauru School of Dentistry, University of São Paulo, São Paulo, Brazil

**Keywords:** Temporomandibular disorders, Myofascial pain, Botulinum toxin type A, Chronic pain, Pain

## Abstract

The antinociceptive effect of BoNT-A have been well documented in animal studies; however, results of few but well-designed randomized placebo-controlled clinical trials about BoNT-A efficacy in masticatory myofascial pain (MFP) are inconsistent. Therefore, the present randomized, double-blind, placebo-controlled clinical trial evaluated the efficacy of BoNT-A in patients with refractory MFP. Twenty-eight patients with pain reduction of less than 30% despite conservative treatment and with an average pain intensity of > 50 mm on the visual analogue scale (VAS) participated. Patients were randomly assigned to receive a total of 80 U of BoNT-A or saline solution (SS) injected into the masseter and anterior temporalis muscles. Pain intensity (VAS), quantitative sensory testing (QST), conditioned pain modulation (CPM), and psychosocial status were examined. Follow-up was performed at 1 and 6 months. For repeated-measure comparisons between evaluation times, Friedman test with Bonferroni correction was used for pain and somatosensory variables and the Wilcoxon test for the psychosocial variables. The Mann–Whitney test was used for all comparisons between groups. The BoNT-A group had a significant decrease in pain intensity at follow-ups compared with the SS group (*p* < 0.001). QST assessment revealed higher pressure pain threshold values in the masseter muscle for BoNT-A group compared to SS (*p* < 0.03) at all follow-ups. No differences were found for mechanical pain threshold and wind-up ratio values (*p* > 0.05) in the entire study. The BoNT-A group presented the most efficient CPM effect (*p* < 0.03) only at the 1 month follow-up in the masseter muscle. There was a significant time effect for BoNT-A in all psychosocial variables (*p* < 0.05) and a drug effect in the Central Sensitization Inventory (*p* < 0.01), Pittsburgh Sleep Quality Index (*p* < 0.004), and Healthy Survey 36 (*p* < 0.05) at 6 months follow-up. The study demonstrates that a single injection-session of BoNT-A has positive effects on the hall pain spectrum of patients with refractory masticatory myofascial pain.

## Introduction

Patients with masticatory myofascial pain (MFP) experience constant and varying degrees of pain and physical disability, neurobiological alterations, psychosocial impairment, and a reduction in well-being (impaired quality of life)^1,2^. MFP accounts for 45% of temporomandibular disorder (TMD) diagnoses^3^ and its management involves a variety of multimodal and reversible-conservative therapies (e.g., counseling, pharmacotherapy, and oral appliances)^4^. This treatment plan is usually effective, but some patients do not improve pain significantly. A 5-years longitudinal study found differences in the time course of MFP: 31% had persistent condition, with low probability of remission^5^. Therefore, refractory MFP requires a treatment plan involving therapies with peripheral and especially central analgesic effects, together with a psychosocial approachment that can improve the entire pain spectrum.

Botulinum toxin type A (BoNT-A) has been considered for treatment of many chronic pain conditions including chronic migraine, neuropathic pain, back pain, and pelvic pain^6–9^. Moreover, it has also been added to the therapeutic array for refractory MFP cases due to its antinociceptive effect. Experimental studies have demonstrated that BoNT-A suppresses the peripheral and central release of neurotransmitters such as glutamate, calcitonin gene related peptide (CGRP), and substance P (SP) when injected into a painful area, thereby reducing peripheral and central sensitization^10–13^. Mechanisms such as the transport to sensory regions of the trigeminal ganglia, modulation of the GABA-ergic system, and reduction of microglia activation may also have an important role in BoNT-A analgesic effects^14–17^. Results from previous research suggest that BoNT-A affects a subset of sensory neurons that responds to inflammatory and mechanical stimulation and is more effective in patients with more severe allodynia^13^. Thus, because refractory MFP is a chronic pain condition, BoNT-A toxin could have positive effects in refractory MFP patients.

Despite the analgesic effects of BoNT-A, the results of few but well-designed randomized placebo-controlled clinical trials (RCT) in MFP are inconsistent^18–23^. Different patient groups and methodologies may explain these differences. Systematic reviews report unclear evidence for the effect of BoNT-A on MFP. However, they recommend considering low doses of BoNT-A in refractory MFP patients to avoid adverse effects on muscle and bone while maintaining analgesic efficacy^24–28^. Most studies on BoNT-A efficacy assessed only improvements in pain intensity, muscle tenderness, mandibular range of motion, and psychosocial impairment. In addition, the Initiative of Measurement Methods and Pain Assessments in Clinical Trials (IMMPACT)^29^ supplemental domains for chronic pain clinical trials recommend the inclusion of biological markers such as quantitative sensory testing (QST), as they could clarify possible pain mechanisms, leading to more effective treatments. MFP patients present different somatosensory profiles compared with healthy controls^1,30^, and because studies on neuropathic pain have shown that BoNT-A regulates somatosensory alterations in this chronic condition, it can be hypothesized that BoNT-A also modulates somatosensory function in MFP patients. However, no evidence of this effect on MFP patients is available^8,31^. Therefore, studies that assess the full pain spectrum and neurobiological mechanism are needed to confirm BoNT-A efficacy for refractory MFP patients.

Therefore, we conducted this study to evaluate the efficacy of BoNT-A over placebo on pain intensity and somatosensory and psychosocial features. The null hypothesis was that the variables studied would not differ between groups and that BoNT-A would not be superior to placebo as an efficacy treatment for refractory MFP patients.

## Methods

This was a single-center, double-blind, placebo-controlled RCT that was conducted at the orofacial pain clinic at the Bauru School of Dentistry from October 01, 2019 to May 16, 2021. The study was approved by the Research Ethics Committee of the Bauru School of Dentistry (CAAE # 79079917.6.0000.5417) and the Brazilian Registry of Clinical Trials (RBR-8fsspyy—15/12/2020) and was conducted according to the guidelines of the Declaration of Helsinki. All subjects were informed about the research purposes and provided written informed consent to participate in this clinical trial. Reporting of data followed the CONSORT checklist for parallel group randomized clinical trials.

### Participants

Patients with TMD of muscular origin treated at or referred to the orofacial pain clinic of the University of Sao Paulo because of refractory myofascial TMD pain were recruited.

Inclusion criteria were age between 18 and 50 years, MFP with and without referral diagnosis according to the Brazilian Portuguese version of the Diagnostic Criteria for Temporomandibular Disorders (DC/TMD)^32^ by two calibrated researchers (kappa coefficient = 0.80) with MFP for more than three months, refractoriness to at least three conservative treatments with pain reduction of less than 30% despite conservative treatment, and an average pain intensity > 50 mm on a 0–100 mm VAS. Exclusion criteria were patients with fibromyalgia, dental and neuropathic pain, traumas to the face and neck, neurological disorders, systemic inflammatory diseases (e.g., arthritis), ongoing orthodontic treatment, use of muscle relaxants and BoNT-A treatment (esthetic or therapeutic), and/or tetanus vaccination in the 3 months prior to study entry.

### Study protocol, randomization, and blinding

**Figure 1:**
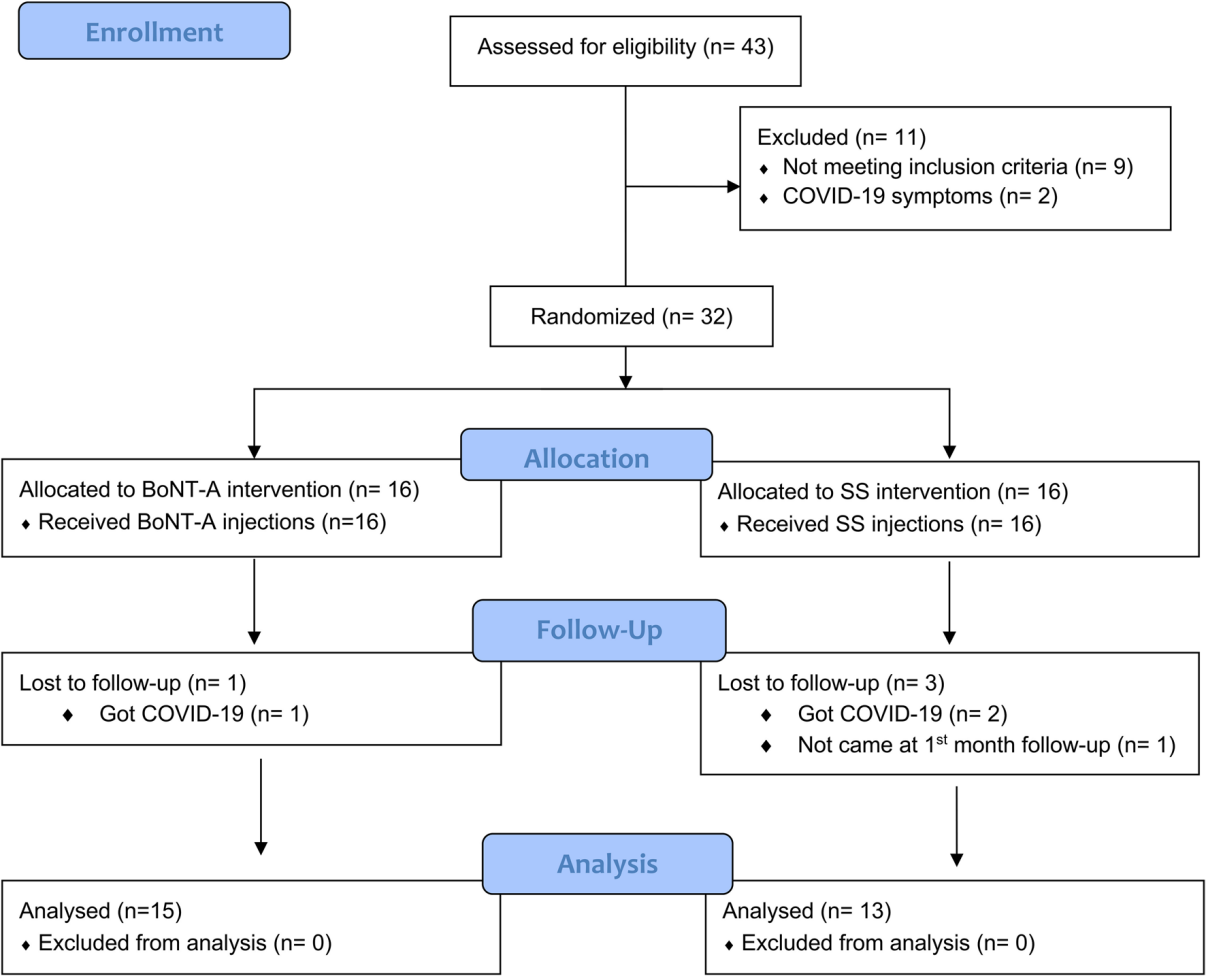
CONSORT flow diagram.

Patients were examined on four occasions. At the first visit, individuals were screened following the inclusion and exclusion criteria of the study. Then, patients who met the criteria and agreed to participate in the study were informed about the treatments and assessment tools used in the study. They were told that they would receive BoNT-A or saline solution (SS) injections in a single session, but that neither the investigator applying the treatments, nor the investigator assessing the outcomes, nor themselves would know which treatment they would receive. BoNT-A and SS are colorless solutions with identical appearance. They were also informed that they should discontinue any TMD treatment from that point onward. One week later, the randomized treatment was administered (visit 2). Follow-up examinations occurred after 1 month (visit 3) and 6 months (visit 4). Figure 1 shows the flowchart of patient enrollment, allocation, and follow-up.

A total of 32 patients were randomized to receive either BoNT-A (n = 16) or SS (n = 16). Randomization was performed using an internet-based computer program (http://www.randomization.com/) in blocks of four patients (block size unknown to the investigators) by a technician who was not involved in any other procedure in the study. The treatment for each patient was recorded and placed in a sealed opaque envelope.

### Interventions

BoNT-A (100 U; Botox, Allergan, Irvine, California, CA, USA) was reconstituted with 1 mL of non-preserved, sterile, isotonic 0.9% saline at room-temperature. A total of 80 U of BoNT-A was used for each patient, divided into 30 U for each masseter muscle and 10 U for each anterior temporalis muscle^23^. A total of 0.8 mL of saline solution served as a control. Bilateral intramuscular injections were performed using a 1 mL syringe with a 30-gauge needle. Subjects were asked to clench their teeth to delineate the muscle area to be injected (masseter and anterior temporalis). After careful aspiration, a total of 5 injections per muscle were applied 5 mm apart. If the aspiration was positive, the needle was moved slightly and aspiration was repeated until it was negative. The patients and the researcher applying the injections were blinded to the treatment. Another researcher not present during injections, opened the sealed envelope, reconstituted BoNT-A and prepared the syringes according to the subjects distribution. Injections were applied in a single session.

### Outcomes

#### Pain variables

Patients rated their pain intensity in the masticatory muscles on a 0–100 mm VAS with the endpoints “no pain” and “worst imaginable pain”^33^. Participants were instructed to make a mark on the VAS indicating the current level of pain (at each visit), the average pain over the last week, and the worst pain over the last month. The mean of the three scores was used for statistical analyses. The changes in average pain scores after treatment served as the primary outcome^34^.

The Portuguese version of the Patient Global Impression of Change Scale (PGIC)^35^ was used to assess the patient’s overall evolution of the treatment outcome. The PGIC has seven subscales assessing pain intensity, disability in activities of daily living, disabilities in social activities, emotions, fear-avoidance, and locus of control behavior. The response options are: no change (or condition has gotten worse); almost the same, hardly any change at all; a little better, but no noticeable change; somewhat better, but the change has not made any real difference; moderately better, and a slight but noticeable change; better, and a definitive improvement that has made a real and worthwhile difference; and a great deal better, and a considerable improvement that has made all the difference. Patients were instructed to select only one alternative at each assessment after treatments.

#### Somatosensory variables

The somatosensory function in TMD patients with MFP has been described in several studies, with patients showing higher sensitivity to mechanical pain stimuli compared to healthy controls^1,36–39^. Therefore, for this study, the following mechanical pain tests of the QST battery were performed: Mechanical Pain Threshold (MPT); Wind-Up Ratio (WUR) (pain summation to repetitive pinprick stimuli), and Pressure Pain Threshold (PPT). Furthermore, pain modulation was assessed using the conditioned pain modulation (CPM) paradigm. Tests were performed on the most painful masseter muscle (trigeminal site) and on the thenar muscle (extra-trigeminal site).

MPT was measured using a standardized set of Semmes–Weinstein monofilaments (Touch-Test TM Sensory Evaluators; North Coast Medical Inc.), which applies forces between 0.008 and 300 g/mm^2^. All pinprick tests were performed with the monofilaments perpendicular to the examination sites, with a contact time of 1–2 s, and an inter-stimulus interval of around 10 s (0.1 Hz). Participants were instructed to verbally report the first sharp or stinging sensation. The limit method was used to determine the threshold using a series of five ascending and five descending stimuli. The MPT was considered as the geometric mean of these ten recordings^40,41^.

To measure the WUR for repeated pinprick stimuli, the same set of Semmes- Weinstein monofilaments was used. For each participant, the monofilament that elicited a numerical response between 3 and 5, indicating pain but not intolerable pain, was selected. The perceived magnitude of a single pinprick stimulus was compared with that of a train of 10 repetitive pinprick stimuli of the same force, applied to an area of 1 cm^2^ at a frequency of 1 Hz. The subject was asked to rate the pain intensity of a single stimulus and the pain at the end of the 10 stimuli using a 0–100 mm VAS with 0 indicating no pain and 100 indicating worst pain imaginable. Single pinprick stimuli were alternated with a train of 10 stimuli until both were done three times. WUR was calculated as the mean score of the three series divided by the mean score of the three single stimuli^40,41^.

PPT was measured with a digital algometer (model Kratos DDK-20) on the surface of the most painful masseter muscle (most prominent part in the functional test) in a relaxed position. The device has a rubber end with a flat circular tip 1 cm^2^ in diameter, through which an increasing and constant pressure of approximately 0.5 kgf/cm^2^ was applied to the test site. Participants were instructed to press a button connected to the algometer at the first painful sensation to interrupt the stimulation. The recordings were repeated three times at 1 min intervals. PPT was determined as the arithmetic mean of the three recordings^40,41^.

To assess the CPM paradigm, as test stimulus (TS), PPT was first obtained for the most painful masseter and for the thenar muscle of the dominant hand. As conditioning stimulus (CS), participants were asked to immerse the non-dominant hand in a container with water at 10 °C for 60 s. The CS pain intensity was maintained between 50 and 70 in a numerical rating scale (NRS). A second assessment of PPT was immediately done after taking the hand out of water at the end of CS. The order of the muscles were the same for all participants. In each evaluation, PPT was determined as the arithmetic mean of three consecutive measurements. The absolute (kgf/cm^2^) and percent (%) differences between PPT values (‘TS before CS’ and ‘TS after CS’) were considered as the CPM values. Negative values indicate an increase in pain threshold^42,43^.

#### Psychosocial variables

To assess the psychosocial status of patients before and after treatment, the validated Brazilian Portuguese translations of the following questionnaires were used: the Hospital Anxiety and Depression Scale (HADS), which^44^ consists of two subscales, one measuring anxiety with seven items and the other measuring depression with seven items scored separately; the Central Sensitization Inventory (CSI), which^45^ contains part A with 25 statements about current health symptoms and part B assessing previously diagnosed central sensitivity syndromes and related conditions; the Perceived Stress Scale (PSS)^46^, which consists of 14 items that measure the perception of stress; the Pittsburgh Sleep Quality Index (PSQI)^47^, which consists of 19 items that measure sleep quality and disturbances; Pain Catastrophizing Scale (PCS)^48^, which consists of 13 items about the degree of thoughts or feelings related to pain; Pain Vigilance and Awareness Questionnaire (PVAQ)^49^, which consists of 16 items that measure attention to pain; and the Short Form Health Survey 36 (SF-36) composed of 36 items, grouped into eight dimensions of health^50^.

Pain and somatosensory variables were assessed at baseline and at 1 and 6 months after treatment. Psychosocial variables were assessed at baseline and at 6 months follow-up.

#### Adverse events

At the 1 month-follow-up appointment the patients were asked to report any adverse events occurring after injections, such as edema, itching, or pain at the injection site, as well as facial assymetries.

### Statistical methods

The G*Power 3.1.9.2 software was used for sample size calculation (Düsseldorf, Germany). The sample size was based on the average pain scores of a previous study^22^. Power calculation showed that nine patients per group would demonstrate more than 90% power when = 0.05 for comparisons between two independent means (T-test). However considering a 30% of possible dropouts, a final number of 12 participants per group was defined as minimum reasonable required sample per group.

All data were entered into a spreadsheet and organized. The Kolmogorov–Smirnov test indicated that the data was not normally distributed. Therefore, for repeated-measure comparisons between the evaluation times, the non-parametric Friedman test with Bonferroni correction was used for pain and somatosensory variables and the Wilcoxon test was used for the psychosocial variables. For most comparisons between groups, the Mann–Whitney test was used. Frequency data, however, were analyzed with chi-square test. All data were analyzed using SPSS Statistics 25.0 software (IBM^®^, New York, NY, USA). A 5% probability level was considered significant in all tests. The proportion of patients with pain reduction (VAS) in both groups was analyzed by Intention To Treat (ITT) analysis. Therefore, it was considered that patients who received treatment but did not attend follow-ups did not experience pain reduction. In total, 32 participants were randomized, 16 for each group. Four participants in the interval between administration of treatment and the first follow-up were loss; three in the SS group and one in the BoNT-A group. The number needed to treat (NTT) was calculated according to the equation $NTT= \frac{1}{ARR}$, in which ARR indicates the absolute risk reduction—that is, the difference between the proportion of patients who did not experience pain reduction in the SS group and the same proportion in the BoNT-A group^51^ NTT was also analyzed by ITT.

## Results

### Patient’s characteristics

Overall, 43 patients were screened; nine (20.9%) failed the inclusion criteria, two were not enrolled due to COVID-19 symptoms, so 32 eligible patients were enrolled in this study (Fig. 1). Nearly all patients completed the study, except for one patient in the BoNT-A (got COVID-19) and three patients in the SS group (two had COVID-19 and one was lost to follow-up).

There were no major differences in demographic characteristics across treatment groups (Table 1). Most patients were women of Latino ethnicity and only four men were enrolled in the study (two in each group). The mean age (standard deviation, SD) was 43.1 (7.1) years. The education level was not high, with 71.4% having only completed secondary school. None of the participants was unemployed. Pain duration ranged from 6 months to 13 years with an average of 4.7 (SD 2.5) years. Twenty patients (71.4%) presented bilateral masticatory muscle pain and 8 (28.5%) presented unilateral masticatory muscle pain. Regarding other DC/TMD diagnoses, five (17.8%) patients presented arthralgia in the temporomandibular joints and 13 (46.4%) had disc displacement with reduction. Migraine or tension headaches (42.8%) followed by irritable bowel syndrome (28.5%) and anxiety or panic attacks (21.4%) were the most prevalent comorbidities of the patients (Table 1).

**Table 1:** Descriptive characteristics of the study population.

Characteristics	BoNT-A	SS	*p*
Age	42.2 ± 5.1	43.4 ± 6.2	0.81
Gender			0.75
Female	13 (86.7)	11 (84.6)	
Male	2 (13.3)	2 (15.4)	
Education			0.62
High school	12 (80.0)	9 (69.3)	
University	3 (20.0)	4 (30.7)	
Occupation			0.82
Student	2 (13.3)	1 (7.7)	
Employed	13 (86.7)	12 (92.3)	
Unemployed	0 (0)	0 (0)	
Pain duration			0.43
1–3 years	5 (33.3)	4 (30.8)	
4–6 years	7 (46.7)	8 (61.5)	
More than 6 years	3 (20.0)	1 (7.7)	
DC/TMD diagnoses			0.50
Myofascial pain with/without referral	6 (40.0)	4 (30.8)	
Myofascial pain/Arthralgia	3 (20.0)	2 (15.4)	
Myofascial pain/Disc displacement with reduction	6 (40.0)	7 (53.8)	
Comorbidities (central sensitization index)			0.10
Restless leg syndrome	1 (6.7)	1 (7.8)	
Chronic fatigue syndrome	1 (6.7)	0 (0)	
Fibromyalgia	2 (13.3)	4 (30.7)	
Migraine or tension headaches	4 (26.7)	7 (53.8)	
Irritable bowel syndrome	3 (20.0)	5 (38.5)	
Multiple chemical sensitivities	0 (0)	1 (7.8)	
Anxiety or panic attacks	3 (20.0)	3 (23.1)	
Depression	2 (13.3)	2 (15.4)	

Data show mean (± SD) or n (%) and were analyzed statistically with t-test and Chi-square test (*p* < 0.05).

*BoNT-A* botulinum toxin type A, *SS* saline solution.

### Pain variables

**Figure 2:**
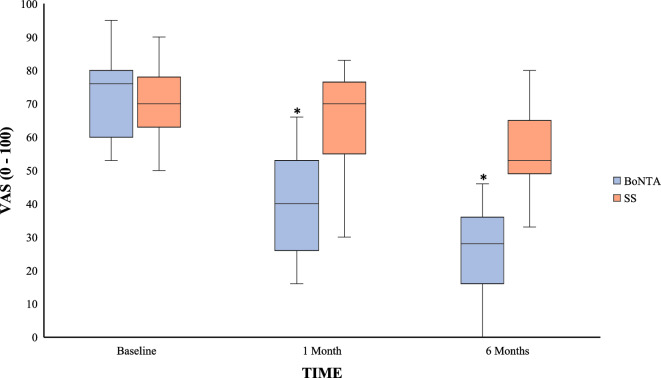
Box plot (median, maximum, minimum 75% percentile, 25% percentile) showing the facial pain intensity (0–100) and inter-group differences assessed at baseline and at one and 6 months after BoNT-A and SS injections. *Inter-group significant differences (Mann–Whitney U-tets, *p* < 0.05).

Baseline data showed no between-group differences in pain intensity (VAS). The median (min–max) pain intensity in the BoNT-A group at baseline was 76 (53–95) and 70 (50–90) in the SS group. After treatment, the pain intensity in the BoNT-A group had a significant decrease to 40 (16–66) and 28 (0–46) at the 1- and 6 months—follow-up. The corresponding figures for the SS-group after treatment were 64 (30–83) and 56 (33–80) for the 1- and 6 months follow-up. The differences between groups were significant at both follow-ups (Fig. 2).

When the proportion of pain reduction was calculated by intention to treat (ITT), 12 patients (75%) in BoNT-A group presented a pain reduction of at least 30% at 1 month-follow-up, versus one patient (6.25% ) in the SS group. Seven patients (43.8%) in BoNT-A group had a pain reduction of at least 50%, versus one patient (6.25%) in SS group. At the 6 months-follow-up, a pain reduction of at least 30% was reported by 15 patients (93.8%) in the BoNT-A group and four patients (25%) in the SS group. Pain reduction of at least 50% was reported by 11 patients (68.8%) in the nBoNT-A group and in one patient (6.25%) in the SS group. The NTT for reducing pain by at least 30% was 1.45 for both the 1- and 6 months- follow-up. The NTT for the reduction of at least 50%, was 2.7 for the 1 month follow-up and 1.6 for the 6 months-follow-up.

**Figure 3:**
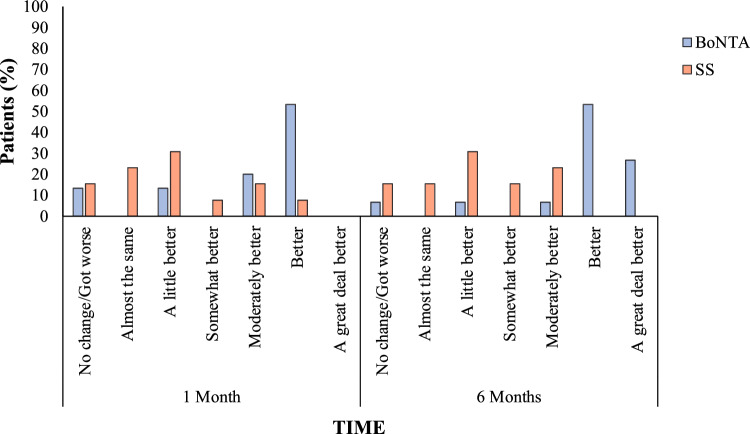
Patient Global Impression of Change Scale assessed at one and six months after treatment with BoNT-A and SS injections. More patients in the BoNT-A group reported improvement at the one and six month follow-up (Chi-square test, *p* < 0.05. *Inter-group significant differences.

Considering PGIC scale scores, even though BoNT-A group presented higher frequencies at the 1 month follow-up in the subscales indicating positive changes after treatment (Fig. 3), there were no significant differences when compared with the SS group (*p* = 0.06). However, at the 6 month follow-up, significant higer frequencies were found in BoNT-A group when compared with the SS group in subscales indicading a greater positive change after treatment (*p* = 0.004).

### Somatosensory variables

At baseline, there were no between-group differences in PPT, MPT, and WUR for the masseter muscle area (Table 2). In the BoNT-A group, a significant increase was found in PPT values in all post-treatment assessments compared to baseline (*p* < 0.03). The between-group comparison showed significantly higher PPT values for the BoNT-A group in post treatment follow-ups (*p* < 0.03). Regarding MPT values, the BoNT-A group presented a significant somatosensory decrease (less sensitive) at the 6 months follow-up compared with baseline (*p* < 0.02) and with the 1 month assessment (*p* < 0.03), but no significant between-group differences were found throughout the study (*p* > 0.05). No time effect or between-drug difference was found in WUR assessment (*p* > 0.05). In the thenar muscle (Table 2), there was no difference in any mechanical pain parameters in the intra- and inter-group comparisons (*p* > 0.05).

**Table 2:** Somatosensory and conditioned pain modulation data of masseter and thenar muscles at baseline, and 1 and 6 months after treatment with BoNT-A or SS.

	Baseline		1 month		6 months	
	BoNT-A	SS	BoNT-A	SS	BoNT-A	SS
Masseter						
PPT	1.0 (0.3–1.8)	1.0 (0.3–1.7)	1.3*^a^ (0.6–2.2)	0.9 (0.4–1.5)	1.3*^a^ (0.6–1.7)	0.7 (0.6–1.8)
MPT	6.3 (0.2–37.3)	9.8 (0.0–60.7)	7.3^#^ (1.2–62.5)	5.9 (1.5–196.3)	14.8* (1.8–62.5)	7.2 (1.5–165.8)
WUR	1.3 (0.9–4.0)	1.3 (0.8–2.4)	1.5 (1.0–2.5)	1.3 (0.8–3.2)	1.4 (1.00–2.4)	1.2 (0.6–2.0)
CPM	− 0.2 (− 1.0 to 0.5)	− 0.0 (− 1.1 to 0.3)	− 0.3^a^ (− 1.1 to − 0.0)	− 0.1 (− 0.67 to 0.14)	− 0.2 (− 1.1 to − 0.1)	− 0.2 (− 1.6 to 0.1)
Difference (%)	− 31.8 (− 101.1 to 28.9)	− 7.1 (− 137.1 to 29.7)	− 23.8 (− 92.3 to − 0.9)	− 15.7 (− 86.1 to 10.0)	− 17.2 (− 78.5 to − 5.7)	− 28.2 (− 95.7 to 6.6)
Thenar						
PPT	2.6 (1.3–4.5)	2.1 (0.9–6.6)	3.0 (2.1–5.1)	2.8 (1.5–7.4)	2.7 (1.6–4.2)	2.7 (1.2–7.6)
MPT	23.9 (4.9–108.5)	77.4 (0.0–232.3)	34.6 (3.1–108.5)	12.6 (0.3–84.3)	41.4 (10.2–134.1)	21.9 (0.3–232.3)
WUR	1.3 (1.0–3.0)	1.4 (0.9–2.0)	1.5 (1.1–2.6)	1.2 (1.0–3.2)	1.2 (1.0–4.1)	1.3 (0.6–2.0)
CPM	− 0.2 (− 1.43 to 1.2)	− 0.4 (− 1.7 to 0.8)	− 0.7 (− 1.5 to 1.4)	− 0.1 (− 1.3 to 0.7)	− 0.8 (− 3.2 to − 0.2)	− 0.4 (− 1.8 to 1.2)
Difference (%)	− 13.5 (− 45.8 to 26.5)	− 13.1 (− 62.1 to 40.3)	− 23.6 (− 57.3 to 43.3)	− 7,6 (− 82,1 to 15.7)	− 31.3 (− 198.5 to − 11.7)	− 17.4 (− 126.7 to 28.7)

Data are presented as median (min–max).

*PPT* pressure pain threshold, *MPT* mechanical pain threshold, *WUR* wind-up ratio, *CPM* conditioned pain modulation paradigm.

^a^Inter-group significant differences.

*Significant differences compared to baseline.

^#^Significant differences between 1 and 6 months follow-up.

In the CPM paradigm, no time effect was found in both groups (*p* > 0.05). At baseline, there were no inter-group significant differences in the masseter and thenar muscles. However, at the 1 month assessment of the masseter muscle, the BoNT-A group had a higher CPM effect (*p* < 0.03) compared with the SS group (Table 2).

### Psychosocial variables

Values for all questionnaires are shown in Table 3. Intra-groups comparisons showed that only the BoNT-A treatment significantly improved the scores of HADS-A/D (*p* = 0.04), CSI (*p* = 0.002), PSS (*p* = 0.006), PSQI (*p* = 0.002), PCS (*p* = 0.02), PVAQ (*p* = 0.02), and SF-36 (1, 2, 3, and 5) (*p* = 0.02) while SS showed no significant changes. Inter-group comparison showed greater values for CSI (*p* = 0.01), PSQI (*p* = 0.004), and SF-36 (3, 4, 5) (*p* = 0.04) after treatment for the BoNT-A group than for the SS group.

**Table 3:** Psychosocial data at baseline and 6 months after treatment with BoNT-A or SS.

	Baseline		6 months	
	BoNT-A	SS	BoNT-A	SS
HADS-A	11.0 (4–15)	6.0 (3–20)	9.0 (3–18)*	10.0 (3–13)
Improbable (0–7)	5 (33.3)	8 (61.5)	7 (46.7)	3 (23.1)
Possible (8–10)	4 (26.7)	4 (30.8)	7 (46.7)	8 (61.5)
Probable (> 10)	6 (40.0)	1 (7.7)	1 (6.7)	2 (15.4)
HADS-D	8.0 (1–12)	5.0 (1–14)	5.0 (0–11)*	7.0 (2–9)
Improbable (0–7)	7 (46.7)	7 (53.8)	10 (66.7)	7 (53.8)
Possible (8–10)	6 (40.0)	5 (38.5)	5 (33.3)	6 (46.2)
Probable (> 10)	2 (13.3)	1 (7.7)	0 (0)	0 (0)
CSI	52.0 (21–84)	46.0 (20–85)	35.0 (15–58)*	51.0 (21–85)^a^
Subclinical (0–29)	2 (13.3)	2 (15.4)	6 (40.0)	1 (7.7)
Mild (30–39)	2 (13.3)	3 (23.1)	4 (26.7)	1 (7.7)
Moderate (40–49)	2 (13.3)	2 (15.4)	3 (20.0)	4 (30.8)
Severe (50–59)	6 (40.0)	3 (23.1)	2 (13.3)	2 (15.4)
Extreme (60–100)	3 (20.0)	3 (23.1)	0 (0)	5 (38.5)
PSS	29.0 (15–38)	24.0 (13–40)	24.0 (14–32)*	27.0 (16–38)
Low (0–13)	0 (0)	1 (7.7)	0 (0)	0 (0)
Moderate (14–26)	5 (33.3)	8 (61.5)	12 (80.0)	6 (46.2)
High perceived (27–40)	10 (66.7)	4 (30.8)	3 (20.0)	7 (53.8)
PSQI	10.0 (4–17)	10.0 (5–16)	7.0 (3–12)*	10.0 (7–16)^a^
Good (0–4)	1 (6.7)	0 (0)	2 (13.3)	0 (0)
Poor (5–10)	8 (53.3)	7 (53.8)	11 (73.3)	8 (61.5)
Sleep disturbance (> 10)	6 (40.0)	6 (46.2)	2 (13.3)	5 (38.5)
PCS	33.0 (12–50)	27.0 (10–47)	27.0 (5–45)*	27.0 (8–45)
PVAQ	51.0 (22–64)	43.0 (32–65)	45.0 (14–57)*	41.0 (31–64)
SF-36				
Physical functioning	70.0 (10–100)	75.0 (25–100)	80.0 (30–100)*	65.0 (10–100)
Limitations due to physical health	50.0 (0–100)	50.0 (0–100)	25.0 (0–100)*	75.0 (0–100)
Pain	41.0 (0–77)	51.0 (31–100)	52.0 (22–84)*	42.0 (22–70)^a^
General health	57.0 (25–97)	72.0 (7–100)	72.0 (27–92)	50.0 (10–82)^a^
Energy/fatigue	45.0 (24–90)	40.0 (10–75	60.0 (30–80)*	45.0 (10–65)^a^
Social Functioning	50.0 (12.5–100)	62.5 (12.5–100)	62.5 (12.5–100)	62.5 (12.5–100)
Limitations due to emotional problems	66.7 (0–100)	100.0 (0–100)	66.7 (0–100)	100.0 (0–100)
Emotional well-being	60.0 (36–92)	44.0 (4–92)	56.0 (36–92)	44.0 (4–92)

Data are presented as median (min–max) score and frequency (%).

*HADS* hospital anxiety and depression scale, *CSI* central sensitization inventory, *PSS* perceived stress scale, *PSQI* pittsburgh sleep quality index, *PCS* pain catastrophizing scale, *PVAQ* pain vigilance and awareness questionnaire, *SF-36* short form health survey 36.

^a^Inter-group significant differences (Mann–Whitney U-test, *p* < 0.05).

*Significant differences compared to baseline (Wilcoxon test, *p* < 0.05).

### Side effects

No patient in any group discontinued the study because of adverse events and no serious adverse events were reported by patients. Minor adverse effects were edema, itching, and pain at the injection site, which are not related to the drug. Regarding mild adverse effects, 10 and two patients, respectively, reported muscle weakness (fatigue) after BoNT-A and SS injections. Two patients in the BoNT-A group reported asymmetric smile and one patient in the BoNT-A group reported difficulty swallowing. All side effects disappeared without intervention.

## Discussion

This randomized, controlled, double-blind study was designed to compare the efficacy of BoNT-A and SS (placebo) for refractory MFP. The study demonstrated the higher efficacy of BoNT-A over placebo at both follow-ups in terms of pain intensity (primary endpoint). Considering the secondary endpoints, BoNT-A was superior only for PPT values in all follow-ups, for CPM values after the 1 month follow-up (masseter muscle), and in CSI, PSQI, and some domains of the SF-36 in the 6 months follow-up. Thus, the null hypothesis was partially rejected.

The effectiveness of BoNT-A in the treatment of refractory MFP was confirmed by consistently significant superiority in pain intensity and in patients’ impression of pain change after treatment. Our results support previous findings from RCTs, three single blinded^18,23,52^ and two double blinded^20,22^ reporting a clear treatment effect of BoNT-A on refractory MFP patients. Nonetheless, two RCTs, crossover double blinded^19,21^, did not find significant improvements with BoNT-A. It is important to point out that some methodologic differences can explain the contrary outcomes. First of all, patients' profiles. Although in the studies of Nixdorf et al.^19^ and Ernberg et al.^21^ the authors describe the sample as a refractory masticatory myofascial pain sample, our study included criteria to classify the individuals as refractory masticatory myofascial pain, once all patients did not respond to at least three conservative treatments with pain reduction of less than 30% for at least 3 months of treatment; also, our study evaluated the “pain chronicity” by a somatosensory point of view, that showed a real impaired of patients internal pain mechanism—scenario in which the BoNT-A toxin is suggested to have more positive effects^10^. Second, differences in injected doses and treated muscles should also be considered. Nixdorf et al.^19^ used 25 U in each temporalis muscle and 50 U in each masseter muscle using three sites per muscle; Ernberg et al.^21^ used 50 U in each masseter muscle in three standardized sites. These doses were significantly higher and less distributed in the muscles that used in our study. The literature suggests that higher doses of BoNT-A are not positively correlated to higher improvement in pain parameters, but only with a possibly higher occurrence of adverse effects such as decreases in the muscle action potential^23^; besides that, considering the actual understanding of central antinociceptive pain mechanism by BoNT-A, a wider distribution of the toxin in the musculature (as done in our study) would be better^13^. Finally, even though the sample size used in the studies could partially explain the different results, the studies designs must be taken into account. The crossover design can bring a disadvantage for clinical studies that compare injectable treatments such as BoNT-A and SS in such a particular condition as masticatory myofascial pain; the literature reports that in this design there may be a carryover effect of one treatment on the other, influencing the results^53^. Thus, the findings of the present study add valuable knowledge, as the available RCTs provide conflicting results.

The IMMPACT recommendations consider treatments with a 30% pain reduction as effective^54,55^. Our study found a 50% pain reduction in the BoNT-A group, but not in the SS group, at the 1 month follow-up, which can be considered a very robust clinical effect. In addition, a 30% and 50% reduction in pain intensity has been shown to be equivalent to “much improved” and ‘‘very much improved” options on the PGIC scale^34^, respectively. Our study observed that a significant larger proportion of BoNT-A subjects, presented with “better” and “a great deal better” in PGIC results, when compared with SS individuals at the 1 month follow-up. This is in line with the study of Guarda-Nardini et al.^56^ which reported a 50% reduction after 1 month of BoNT-A treatment and a 5% reduction after SS. Similarly, Ernberg et al.^21^ and Kurtoglu et al.^20^ found respectively 30% and 22% pain reduction after 1 month of BoNT-A injections, but none and 13%, respectively, after saline injections. However, their findings were not as robust and clinically meaningful compared to ours since they incuded fewer patients.

Animal studies have demonstrated that BoNT-A injections into painful areas, inhibit the release of neurotransmitters such as glutamate, SP, and CGRP and pro-inflammatory cytokines such as IL1-beta and TNF-alpha from nociceptive nerve endings and trigeminal ganglia in chronic pain models^11,12^. Besides, evidence shows that BoNT-A has a central effect on pain as it reaches the central nervous system (CNS) via axonal transport (retrograde and/or anterograde)^57,58^, where a central inhibition of neurotransmitters and nociceptor transduction also occurs^13–15^. Additionally, BoNT-A blocks the release of neuroactive substances from glial cells (astrocytes and microglia), which can contribute to the analgesic activity of BoNT-A^59^. Attal et al.^8^ demonstrated, using biopsies in a neuropathic pain RCT, that pain improvement with BoNT-A was independent of peripheral neurotransmitter levels, which did not change despite BoNT-A injection. The study suggested that despite that peripheral mechanisms could not be ruled out, the main analgesic mechanism of BoNT-A in chronic pain conditions is related to central pain transmission. This mechanism can be the main explanation for the pain improvement in our patients, since we also studied patients with a chronic pain condition.

MFP patients have somatosensory abnormalities, especially in mechanical pain tests^39^. To the best of our knowledge, this was the first study to describe changes in somatosensory and endogenous analgesic system function in refractory MFP patients after BoNT-A treatment, using only mechanical pain tests, which had been previously reported as significant and important in TMD patients among all QST battery^1,36,38,39^. Our study found that patients in the BoNT-A group had significantly higher PPT values in the masseter muscle at all follow-ups compared with the SS group. This result is similar with that of other studies showing a decrease in muscle tenderness to palpation and an increase in PPT values^23^ after 3 and 6 months of BoNT-A treatment compared to placebo. In contrast, Ernberg et al.^21^ reported no differences in PPT between BoNT-A and placebo groups after 3 months. Our study also found that BoNT-A has a positive effect in MPT values, since patients in the BoNT-A group were less sensitive to pinprick stimuli after 6 months of treatment, while no significant differences were found in the SS group during the entire study. Increased sensitivity to pressure and pinprick stimuli has been demonstrated in MFP^38,60,61^ studies. PPT and MPT are mainly mediated by A-delta fibers and in a less proportion by C-fibers, depending of frequencies and intensities of the stimuli^40,62^. Since BoNT-A blocks neurotransmitter release from these fibers, a plausible explanation for the positive effects of BoNT-A in these mechanical tests could be that BoNT-A decreases sensitization of primary nociceptive afferents in the painful area and central hyperexcitability in the trigeminal system, which are known to produce hyperalgesia^61^.

TMD patients have been shown to have impaired CPM^63–65^. Therefore, the effects of BoNT-A on CPM were also evaluated. Our findings showed that patients in the BoNT-A group had more effective CPM in the trigeminal area compared with SS at the 1 month follow-up. Using rat models, Drinovac et al.^16,66^ suggested that the modulation of the opioidergic and GABA-ergic systems by BoNT-A is involved in the central antinociceptive effect of BoNT-A. In addition, the analgesic action in the trigeminal region (nucleus caudalis) involves interactions with the central endogenous opioid system^67^, implying that BoNT-A can also have effects in reestablishing effective pain modulation resulting in the reduced pain sensitivity found in the present study.

It is important to note that there was no time and treatment effect in any mechanical pain test and in the CPM paradigm in the thenar muscle. This result may suggest that BoNT-A cannot reach distant areas when injected into the trigeminal region. However, rat models of polyneuropathy and bilateral neuropathy showed that BoNT-A reduced pain on both the ipsilateral and contralateral sides, excluding passive spread of the toxin and demonstrating systemic spread^14,68^. On the other hand, this result could be related to the fact that the pain process was localized and not due to the effect of the toxin.

In general, TMD patients experience psychosocial distress and have a high prevalence of psychologic disorders. Systematic reviews have reported that between 21% and 60% of TMD patients show depressive symptoms^69^ and 28% to 76% show somatic symptoms^69^. In addition, TMD patients were found to have higher levels of anxiety and catastrophizing, poorer sleep quality, and lower quality of life than the general population^70–73^. Our study found a positive effect of BoNT-A treatment on some assessed psychosocial variables; however it is important to report that even though no significant differences were found at baseline, BoNT-A group presented higher values, making the positive effects of this treatment in the follow-ups more notable. In agreement with our previous study^74^ we found a positive effect of BoNT-A on depression scores also in this study, even if there were no significant differences to the control group. In contrast, Ernberg et al.^21^ did not find any improvement on depression after BoNT-A injections. Improvement in depression symptoms with BoNT-A has also been reported in neuropathic pain and migraine studies^75,76^. These findings and our results indicate that the effects of BoNT-A on psychological symptoms is mainly related to pain relief. However, a migraine study suggests that BoNT-A could ameliorate psychosocial impairment by modulating the central nuclei of the limbic system or by improving patients’ self-esteem as a result of the esthetic effects of BoNT-A in the glabellar region^76^. An intriguing fact is that clinical studies have suggested as an hypothesis, that the injection of BoNT-A in the glabellar region is related to a sustained mitigation of depressive symptoms that did not improve with previous antidepressant medication, by disrupting proprioceptive facial feedback that reinforces negative emotions^77–80^. The exact mechanism by which BoNT-A improves depressive symptoms, however, is unknown. It is quite difficult to compare our psychosocial results with others, since studies assessing the effects of BoNT-A in psychosocial factors are scarce. The improvement of the other psychosocial variables after BoNT-A may be mainly related with the analgesic effect of BoNT-A. It is noteworthy that we found that BoNT-A, improved central sensitization symptoms, which could also support the central analgesic effect of BoNT-A and the indication of BoNT-A in chronic conditions such as refractory masticatory MFP. Finally, these results could had been influenced by the level of education of our sample. Since our sample was mainly composed of participants that presented an acceptable level of education, a greater self-care and motivation regarding the treatment was expected through all the study. In addition, the literature correlates education levels with psychossocial possitive predisposition to seek specialized help for painful conditions^81^.

Some limitations of this study should be discussed. First, we must consider that even though the sample size was calculated based on the primary outcome, the number of patients was small to find differences in the secondary outcomes. Second, although the double-blind design of our study strengthens our results, it was not a multicenter study and it included only a specific group of patients, so caution is recommended when extrapolating our findings. Third, blinding of the study could have been compromised due to BoNT-A aesthetics effects (muscle thickness diminution) and paralysis, despite that injections were for therapeutic purposes. Fourth, we did not assess adverse effects of BoNT-A injections on muscle and bone. The literature indicates that these adverse effects depend on the dose and number of BoNT-A applications^25^. However, our study used doses that produced reversible adverse effects on muscle and no adverse effects on bone in a previous clinical trial conducted by our group^23^. Even though a recent clinical trial showed that the BoNT-A doses used in this study had long-lasting analgesic effects (6 years)^82^, we recommend that the long-term analgesic effects of a single BoNT-A injection be assessed in a RCT to prevent reapplications and thus adverse effects. Fifth, it is important to mention that previous positive experience with BoNT-A treatment could have influenced the results in the BoNT-A group, since we excluded patients only if they had received BoNT-A treatment during the 3 months preceding the study. However, we did not ask about previous BoNT-A treatment beyond that time frame so we do not know if any patient included indeed had received BoNT-A treatment earlier. Finally, we recommend assessing the phenotype of patients who respond positively to BoNT-A treatment.

## Conclusion

Therefore, based on the positive effects of BoNT-A on pain intensity, somatosensory function, and psychosocial status, we conclude that BoNT-A is effective in treating patients with refractory masticatory MFP.

## Data Availability

The datasets generated during and/or analyzed during the current study are not publicly available but are available from the corresponding author on reasonable request.
